# Relation Between Social Support Received and Provided by Parents of Children, Adolescents and Young Adults With Cancer and Stress Levels and Life and Family Satisfaction

**DOI:** 10.3389/fpsyg.2022.728733

**Published:** 2022-02-07

**Authors:** Anabel Melguizo-Garín, Mª José Martos-Méndez, Isabel Hombrados-Mendieta, Iván Ruiz-Rodríguez

**Affiliations:** Department of Social Psychology, University of Malaga, Málaga, Spain

**Keywords:** social support received, social support provided, parents of children with cancer, stress levels, family satisfaction, life satisfaction

## Abstract

**Introduction:**

The present study aims at analysing how social support received and provided by parents of children, adolescents and young adults (AYA) diagnosed with cancer, as well as their sociodemographic and clinical variables, affect those parents’ stress levels and life and family satisfaction.

**Materials and Methods:**

A total of 112 parents of children and AYAs who had been diagnosed with cancer and who received treatment in Malaga participated in the study. In the study, participated all parents who voluntarily agreed to fulfil the questionnaire. The main inclusion criterion was that their child had cancer. Instruments used were Questionnaire on the Frequency of and Satisfaction with Social Support (QFSSS), Paediatric Inventory for Parents (PIP), Life Satisfaction Scale and Family Satisfaction Scale.

**Results:**

In the mean difference analyses, male parents showed 3.38 (SD = 0.56) in social support received and female parents showed *M* = 3.08 (SD = 0.72). Conversely, in social support provided, female parents showed 3.22 and male parents showed *M* = 3.55 (*p* = 0.020). Significant differences were also found in family satisfaction, where female parents (*M* = 3.64) feel more satisfied than male parents (*M* = 3.06; *p* = 0.027). Parents of children aged between 0 and 14 years (*M* = 3.06) feel more stress than those parents of children aged 15–21 (*M* = 2.61; *p* = 0.021). The correlation analysis shows that there is a negative and significant relation between stress levels experienced by parents when facing different situations related to the child’s disease and both types of support, received *r* = −0.411, *p* < 0.001 and provided *r* = −0.282, *p* < 0.01. There is also a positive and significant relation between life satisfaction and social support received *r* = 0.292, *p* < 0.01, and social support provided *r* = 0.409, *p* < 0.001. There is a positive and significant relation between family satisfaction and social support received *r* = 0.330, *p* < 0.01, in the same way as with social support provided *r* = 0.222, *p* < 0.05. The regression analysis related to stress levels of parents indicates that social support received predicts levels of stress significantly *p* < 0.001, with the variable of number of children being the one that showed to be significant *p* < 0.05. Social support provided showed the most significant results *p* = 0.001, meaning that social support provided increased life satisfaction. Social support received explains family satisfaction (*p* = 0.50), as it increases the family satisfaction of parents of children with cancer.

**Discussion:**

Analysing social support received and provided, as well as sociodemographic and clinical variables, allowed us to broaden the knowledge on the effect social support has on stress levels, life satisfaction and family satisfaction in parents of children and AYAs diagnosed with cancer. This may have relevant practical implications for the design of interventions that would improve parents’ lives.

## Introduction

Cancer is a disease that affects the lives of patients and their relatives both socially and psychologically (Molinaro and Fletcher, 2018; Carlsen et al., 2019; Wilford et al., 2019) and it is a great challenge for those families whose members diagnosed with cancer are minor CHILDREN OR ADOLESCENTS (Van Schoors et al., 2017; Sánchez-Egea et al., 2019). We refer to childhood cancer that includes ages 0 to 14. In this age range, minors are treated by a specialised service of paediatric oncology. In recent years, reference has begun to be made to childhood-teenage cancer, including the ages of 15 to 21. Traditionally, patients within this age group would receive adult oncology services; however, increasingly more units, services and hospitals are considering adolescents and young adults (AYA) between 15 and 21 to be closer to the paediatric oncology approach, thus understanding them with their own characteristics and particularities. This approach also aims at applying a more appropriate transition from teenage to young adulthood and hence provide better services to these groups (Ministerio de Sanidad, Servicios Sociales e Igualdad de España, 2015). Families facing childhood cancer must deal with a wide range of situations (Enskär et al., 2020), which include high frequency of treatments and hospitalisation of the minor, side effects associated with such treatments, uncertainty about the course of the disease and fear of a possible relapse (Sloper, 2000; Ångström-Brännström et al., 2018). These situations contribute to higher levels of stress in parents (Hoven et al., 2017; Tillery et al., 2020). In fact, many parents have shown symptomatology linked to stress even after their children overcome the disease (Norberg and Boman, 2013; Bilodeau et al., 2018). The situations linked to most of the stress parents suffer are related to hospitalisations (Liu et al., 2020), receiving information related to the child (Smith et al., 2019) and waiting times in diagnoses and tests (Patterson et al., 2003).

There are some protective factors against stress. One of the most relevant is receiving social support, which makes it possible for parents not to experience the same psychological effects over time (Schirren and Boman, 2010). The study of social support is becoming a field of special interest in psycho-oncology (Herrero and Gracia, 2007; Gunter and Duke, 2019), as it provides information on how patients and their relatives cope with the disease. More precisely, social support has proved to reduce stress levels in patients (Sloper, 2000). Support is understood as an exchange of aid provided by one person to another, as well as the social resources that individuals perceive as available at a given time (Gottlieb and Bergen, 2010). Support relates to feeling valued and cared for as part of a social network of mutual support (Taylor et al., 2004).

Very few studies analyse social support taking into consideration its double dimension, that is, satisfaction with social support received and satisfaction with social support provided to others. The same person may provide support and receive it at the same time. It is therefore important to know the impact of this double function of support in variables, such as stress and life and family satisfaction of parents of children with cancer (Sloper, 2000; Carlsen et al., 2019). Social support should be analysed in a multidimensional way (Melguizo-Garín et al., 2019); however, most studies only analyse the social support that parents receive from their networks. Studies like the one by Tremolada et al. (2012) show that the perception of social support that mothers of children with leukaemia receive relates to less psychological symptoms and higher satisfaction with life. Other authors suggest that perceiving support could be related to feeling less distress caused by the child’s diagnosis (Hoekstra-Weebers et al., 2001). Jou and Fukada (2002) note that for support to be beneficial, there must be a balance between what is given and what is received. When individuals provide more support than they receive, they may feel an excessive burden. Conversely, when individuals receive more support than they provide, self-esteem decreases and there is a feeling of being a debt. Jaeckel et al. (2012) also found that the lack of reciprocity in social support could have negative effects on wellbeing. Despite this dimension of social support provided having been scarcely studied, the balance between support received and support provided is key for the life satisfaction and health of individuals as it is shown by the studies mentioned above.

Cancer has a significant impact on life satisfaction and parents’ family satisfaction. Parents’ satisfaction is one of the variables that is most affected in the process of childhood cancer (Faith et al., 2019). Different situations related to the child’s treatment and the alteration of family routines are sources of stress and can also lead to low life satisfaction (Espada and Grau, 2012). Social support has a positive effect on parents’ life satisfaction (Gibbins et al., 2012; Marsland et al., 2013). Another area that has been proved to be affected in parents of children with cancer is family satisfaction. When childhood cancer appears, parents focus on caring for the ill child, which can have a negative impact on family life, family satisfaction and the quality of family relations (Salvador et al., 2019). This notion is deeply determined by individual, relational and social factors (Acevedo et al., 2007). Family satisfaction is understood as the interactional wellbeing of the family members (Sobrino, 2008). Low family satisfaction resulting from family difficulties due to the child’s disease is related to lower quality of life (Salvador et al., 2019). Cohesion between family members is tightly linked to family satisfaction and positive adaptation to the situation is negatively linked to stress suffered by parents (Yoon et al., 2018). Authors, such as Wasserman and Danforth (1988), consider that social support constitutes a family phenomenon and a shared place of interaction between individuals. It seems that family can be considered as a space where its members relate spontaneously with one another and in which non-problematic events as well as those events that jeopardise the family balance must be dealt with. There is little research on how social support relates to the family satisfaction of those parents whose child has cancer, even more if we consider the double functionality of social support (received and provided).

The present study aims at analysing how receiving and providing support by parents of children with cancer affects stress levels and life and family satisfaction. The variable of family satisfaction in the context of childhood cancer has been scarcely studied. One of the new contributions of this study is that social support is analysed considering two dimensions, social support received by parents of children with cancer and social support provided by them (to their network and close family). There is little research on the effect of social support received and provided in the context of families with children with cancer and its relation to life and family satisfaction, as well as stress levels.

Considering the objectives of the study, the following hypotheses are suggested as:

1. There is a negative relation between satisfaction from social support received and provided and stress levels.
2. There is a positive relation between satisfaction from social support received and provided and life and family satisfaction.
3. Higher levels of satisfaction from social support received and provided predict lower levels of stress and higher life and family satisfaction.

## Materials and Methods

### Participants

A total of 112 parents of children and AYAs with cancer who received treatment at the Children’s Hospital of Málaga (Spain) participated in the study. Participants were selected based on their voluntary wish to participate. Participants were at different stages of their children’s cancer disease and treatment. The sample was gathered from parents of children and adolescents who received treatment at the Mother and Child Hospital of Malaga and from parents of patients aged between 15 and 21 from the Regional Hospital of Malaga (Spain). Once parents were explained the aim of the study and what the procedure would be like, those who decided to participate in the study formed the final sample. Inclusion criteria for the study sample were the following: parents or legal guardians of patients aged from 0 to 21 with cancer disease. Conversely, exclusion criteria were the following: other relatives of patients who were not the parents or legal guardians and parents whose child had deceased. The sociodemographic questionnaire did not include questions about the stage of treatment (on-going or follow-up). However, all participants attended the Hospital for their children to receive treatment related to cancer (follow-up consultations, ambulatory treatment, hospitalisation, etc.).

### Procedure

Parents who participated in the study went regularly to the Children’s Hospital of Malaga. They were contacted directly at the hospital (oncology rooms, semi—private accommodation and hospitalisation area) or in some rooms for children with cancer that the local NGO has within the hospital used to provide support to families of children with cancer. Participants received a written and verbal informed consent about the procedure that would be carried out. The study was approved by the Ethical Committee on Scientific Research from the Regional Government of Andalusia (Spain), CEI 2017. Once participants had signed the informed consent, they were given the option to choose between two ways of completing the instrument: on paper during some of their visits to the hospital or online through a digitalized version of the instrument, which was anonymously and automatically added to a database upon completion.

### Measures

#### Sociodemographic Questionnaire

The sociodemographic questionnaire included questions related to gender, age and marital status of participants (level of qualifications, employment situation, number of children and number of people under their care). This questionnaire also includes questions about the child: gender, type of cancer and length of time since diagnosis.

#### Questionnaire on the Frequency of and Satisfaction With Social Support

Questionnaire on the Frequency of and Satisfaction with Social Support (QFSSS) by García-Martín et al. (2016) was used. This questionnaire was used to measure social support received and provided by parents. More precisely, we measured the type of support (emotional, instrumental and informative) provided by the different sources of support from parents’ social networks (partners, relatives, friends and members of the community and the association), as well as the type of support provided by parents to the different sources. The questionnaire comprises 12 items on support received and 12 items on support provided. Answer options for each dimension are five, where ‘1’ means never and ‘5’ means always in terms of frequency and ‘1’ means unsatisfied and ‘5’ very satisfied in terms of satisfaction. This study uses the average score of social support received considering the three types of support and the four types of sources, as well as the average score of social support provided considering the same three types and four sources of support. Cronbach’s Alpha of the full scale is *α* = 0.96. The following are some examples of the items included in the satisfaction with social support received section: ‘*you receive care and affection and you are listened to when you need to talk and express emotions* (emotional support from partner) and ‘*He/she is willing to do things for you, such as helping you with your daily chores or in the care of your child*’ (functional support), and these are some examples of items included in the satisfaction with social support provided section: ‘*you give them useful advice and information to solve their doubts, problems or daily chores*’ (informational support provided to friends) and ‘*You are willing to do specific things for them, such as helping them with their daily chores or care’* (informational support provided to community).

#### Paediatric Inventory for Parents

Paediatric Inventory for Parents (PIP), by Streisand et al. (2001), was used, in its adaptation and validation from Del Rincón et al. (2007), Spanish version. This questionnaire measures levels of stress caused by situations parents of children with cancer face daily. It comprises a frequency scale and an effort scale. The following are two examples of items included in the frequency scale and the effort scale, respectively: ‘*How often do you experience sleeping problems?’* and ‘*How difficult is it for you to attend your child’s medical tests and treatments*’. Each scale comprises 42 questions related to situations faced by parents during their child’s disease. Participants must answer how frequently each situation occurs and must choose between five options, where ‘1’ means never and ‘5’ means very often. Subsequently, they must also answer according to the effort such situations take, where ‘1’ means none and ‘5’ means very much. Cronbach’s Alpha of the full scale is *α* = 0.95.

#### Life Satisfaction Scale

Life Satisfaction Scale from Pavot and Diener (1993) was used. This scale offers a general index of life satisfaction, where life satisfaction is understood as a general construct of subjective wellbeing. It is a unidimensional scale comprising five items, which are answered through a Likert-type scale of 7 points (1 = completely disagree and 7 = completely agree). The following is and an example of an item in this scale: *‘In most things, my life is very close to how I want it to be’*. Cronbach’s Alpha of the full scale is *α* = 0.87.

#### Family Satisfaction Scale

Family Satisfaction Scale from Olson and Wilson (1982) was used, in its translated and adapted version. The scale was reduced to 10 items (Olson and Wilson, 1982), which measure the level of satisfaction in relation to family cohesion and adaptability. Answers to the Family Satisfaction Scale range between ‘1’ (very unsatisfied) and ‘5’ (very satisfied). This study uses the total average score obtained by calculating the average of all scores and then unifying both dimensions, family cohesion and adaptability. The following are some examples of items included in this questionnaire: ‘*My family’s ability to share positive experiences’ and* ‘*the quality of the communication between the family members’*. Cronbach’s Alpha of the full scale is *α* = 0.95.

### Data Analyses

Statistical analyses were carried out through SPSS (v.25). No missing values were found. Data met the criteria to carry out the suggested analyses and there was no collinearity between the study’s variables. First, sociodemographic and clinical characteristics of the study’s sample were analysed, and then, a descriptive analysis of the study’s variables was carried out. Mean differences were also analysed to verify if there were significant differences between results of the study’s variables in the sample based on parents’ gender, children’s and AYAs’ age and time from diagnosis. In order to know the relation between the different variables (clinical and sociodemographic variables of the sample, social support received and provided, stress and life and family satisfaction), the Pearson’s correlation coefficient was calculated. Finally, a multiple linear regression analysis was carried out to find out more about the existing relations between the variables of the study. Three regression models were carried out as: one for stress, another for life satisfaction and a third one for family satisfaction. These variables acted as dependent variables. Independent variables in the three models suggested were as: social support received and social support provided, and the sociodemographic and clinical variables of the sample. The variables considered for the regression equation were those with statistical relevance (*p* < 0.05).

## Results

### Demographic and Clinical Characteristics of the Study Cohort

The sample comprised 33.9% men and 66.1% women, with an average age of 41 years (SD = 6.93). Most participants were married or lived with their partners (88.5%). The remaining were single, divorced or widowed (11.5%). Regarding the gender of the children, 58% were boys and 42% girls, with an average age of 8 years (SD = 5.02). Regarding the type of cancer, 54.5% suffered from leukaemia, 9% from Ewing sarcoma, 8% from lymphoma, 4.5% from medulloblastoma and the remaining suffered from other types of childhood cancer. The length of time from diagnose was the following: 18.9% of children had been diagnosed less than 1 year before, 23.4% 1 year, 19.8% 2 years ago, 9.9% 3 years ago, 12.6% 4 years ago and the remaining 15.3% 5 or more years ago. All data can be seen in Table 1.

**Table 1:** Sociodemographic variables (*n* = 112).

Parents	
Age of parent/guardian	41(6.93)^a^
Number of children	1.98(0.67)^a^
Number of people under care^*^	2.18(1.15)^a^
Age of child with cancer	8(5.02)^a^
	%(*N*)
Age of child 0–14 years	87.5(98)
Age of child 15–21 years	12.5(14)
Gender of the parent/guardian	
Male	33.9(38)
Female	66.1(74)
Marital Status	
Single	3.6(4)
Married	80.4(90)
Divorced	3.6(4)
Separated	3.6(4)
Widowed	0.9(1)
Unmarried partner	1.8(2)
Living as a couple	6.3(7)
Education Level	
University degree	31.3(35)
Vocational Training	33(37)
A Levels	11.6(13)
Secondary Education	22.3(25)
Other	1.8(2)
Employment Situation	
Civil Servant	13.4(15)
Self-employed	11.6(13)
Employee	31.3(35)
Unemployed	33(37)
Domestic work	10.7(12)
Children	
Gender of the child with cancer	
Boy	58(65)
Girl	42(47)
Type of cancer	
Leukaemia	53.6(60)
Ewing Sarcoma	8.9(10)
Lymphoma	8(9)
Medulloblastoma	4.5(5)
Neuroblastoma	4.5(5)
Rhabdomyosarcoma	2.7(3)
Hepatoblastoma	2.7(3)
Astrocytoma	1.8(2)
Other	13.4(15)
Length of time since diagnose	
Less than 1 year	18.9(22)
1 year	23.4(26)
2 years	19.8(22)
3 years	9.9(11)
4 years	12.6(14)
5 or more years	15.3(17)

a *Average (Deviation)*.

* *Carers of the elderly, dependent persons*.

### Descriptive Characteristics of the Outcomes

To know participants’ stress levels, their social support received and provided and their life and family satisfaction, descriptive analyses were carried out, as it can be seen on Table 2. Results indicate that parents express feeling medium-high levels of stress when they must face situations related to their child’s disease *M* = 3.01, SD = 0.67. They report receiving medium levels of support *M* = 3.18, SD = 0.68 and providing medium-high support *M* = 3.33, SD = 0.70. They also express having medium-low levels of life satisfaction *M* = 3.52, SD = 1.17 and medium-high levels of family satisfaction *M* = 3.48, SD = 0.90. The reliability of each instrument used in this study can be observed in Table 2. This was calculated through Cronbach’s alpha.

**Table 2:** Mean and standard deviation of social support received and provided, stress and life and family satisfaction.

	*N*	*M*	SD	*Reliability*
Support received	112	3.18	0.68	0.92
Support provided	112	3.33	0.70	0.95
Stress	112	3.01	0.67	0.95
Life satisfaction	112	3.52	1.17	0.87
Family satisfaction	112	3.48	0.90	0.95

### Mean Differences

To find out whether there are significant differences between parents according to gender, the age of their ill children (and if these children belonged to the group 0–14 years or 15–21 years) and according to the time passed from the diagnosis, *T*-tests on independent samples were carried out. Through these tests, the aim was to know the differences in the variables of social support received, social support provided, stress, life satisfaction and family satisfaction. Results from analyses are shown below.

The *T*-test with participants’ gender as grouping variable showed significant differences when it comes to satisfaction with social support received and social support provided. For social support received, male parents show a mean of 3.38 (SD = 0.56) and female parents *M* = 3.08 (SD = 0.72) with *p* = 0.015. Regarding social support provided, female parents show a mean of 3.22 (SD = 0.73) and male parents *M* = 3.55 (SD = 0.61) with *p* = 0.020. This means that fathers feel more satisfied with the support they provide and receive than mothers. Significant differences were also found in family satisfaction, where female parents showed *M* = 3.64 (SD = 0.84), thus meaning they feel higher family satisfaction than male parents *M* = 3.06 (SD = 0.96) with *p* = 0.027. Regarding stress and life satisfaction, no significant differences were found based on participants’ gender (Table 3).

**Table 3:** Mean differences in social support, stress, life satisfaction and family satisfaction according to the gender of the parent.

	Social support received		Social support provided		Stress		Life satisfaction		Family satisfaction	
	*M*(SD)	*p*	*M*(SD)	*p*	*M*(SD)	*p*	*M*(SD)	*p*	*M*(SD)	*p*
Gender										
Male	3.38(0.56)	0.015	3.55(0.61)	0.020	2.85(0.68)	0.076	3.80(0.99)	0.081	3.06(0.96)	0.027
Female	3.08(0.72)		3.22(0.73)		3.09(0.66)		3.39(1.24)		3.64(0.84)	

The *T*-test with children’s age as grouping variable showed significant differences according to the stress variable. Parents of children aged between 0 and 14 *M* = 3.06 (SD = 0.66) experience more stress than those parents whose children are aged between 15 and 21 years *M* = 2.61 (SD = 0.68) with *p* = 0.021. Regarding the remaining variables (social support received, social support provided, life satisfaction and family satisfaction), no significant differences were found based on children’s ages (Table 4).

**Table 4:** Mean differences in social support, stress, life satisfaction and family satisfaction according to the age range of the child.

	Social support received		Social support provided		Stress		Life satisfaction		Family satisfaction	
	*M*(SD)	*p*	*M*(SD)	*p*	*M*(SD)	*p*	*M*(SD)	*p*	*M*(SD)	*p*
Age range of the child										
0–14	3.19(0.68)	0.677	3.34(0.71)	0.628	3.06(0.66)	0.021	3.52(1.20)	0.991	3.52(0.93)	0.390
15-over	3.11(0.70)		3.25(0.64)		2.61(0.68)		3.53(1.01)		3.18(0.54)	

Lastly, a *T*-test was carried out with time since diagnosis as grouping variable. As it can be seen on Table 5, no significant differences were found when testing this variable with the study’s variables (social support received, social support provided, stress, life satisfaction and family satisfaction).

**Table 5:** Mean differences in social support, stress, life satisfaction and family satisfaction according to the diagnosis time.

	Social support received		Social support provided		Stress		Life satisfaction		Family satisfaction	
	*M*(SD)	*p*	*M*(SD)	*p*	*M*(SD)	*p*	*M*(SD)	*p*	*M*(SD)	*p*
Diagnosis time										
0–2 years	3.27(0.63)	0.102	3.30(0.65)	0.494	3.00(0.64)	0.891	3.66(1.10)	0.123	3.49(0.84)	0.971
3-over years	3.05(0.75)		3.40(0.79)		3.02(0.74)		3.30(1.28)		3.48(1.03)	

### Univariate Analysis

Existing relations between support received and provided by parents and stress and life and family satisfaction are described in this section. Pearson’s correlation coefficients were calculated, as it can be seen in Table 6. Results show there is a negative and significant relation between stress levels experienced by parents when facing different situations related to the child’s disease and both types of support, received *r* = −0411, *p* < 0.001 and provided *r* = −0.282, *p* < 0.01. There is also a positive and significant relation between life satisfaction and social support received *r* = 0.292, *p* < 0.01, and social support provided *r* = 0.409, *p* < 0.001. Likewise, a positive and significant relation was also found between family satisfaction and social support received (*r* = 0.330, *p* < 0.01), and social support provided (*r* = 0.222, *p* < 0.05.). No significant relations were found between sociodemographic and clinical variables and stress, life satisfaction and family satisfaction.

**Table 6:** Correlations between social support received and provided and clinical and sociodemographic variables with stress, life and family satisfaction.

	Stress	Life Satisfaction	Family Satisfaction
Social support received	−0.411^***^	0.292^**^	0.330^**^
Social support provided	−0.282^**^	0.409^***^	0.222^*^
Age of parent/guardian	−0.112	0.002	−0.305^*^
Number of children	−0.188	0.055	0.124
Age of child with cancer	−0.059	−0.079	−0.231
Diagnosis time	−0.101	−0.008	−0.063

* *p < 0.05;*

** *p < 0.01; and*

*** *p < 0.001*.

### Multivariable Analysis

A multiple linear regression analysis was carried out on three models suggested. Models can be seen further down. Social support received and provided and the sociodemographic and clinical variables were considered independent variables in each model; dependent variables were stress, life satisfaction and family satisfaction.

**Figure 1:**
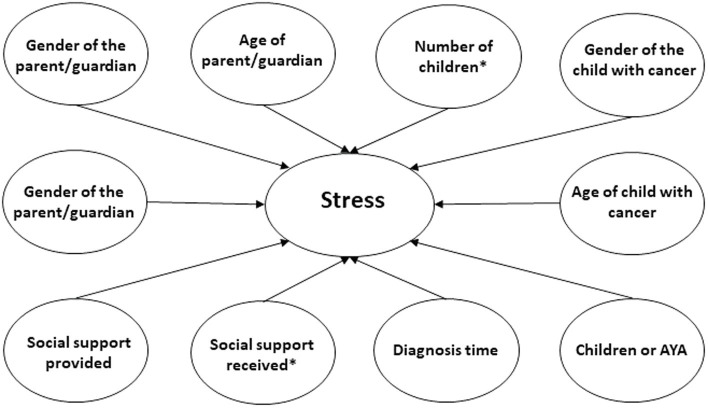
Stress model.

For the first model analysed, parents’ stress was considered dependent variable (Figure 1). The regression analysis related to stress levels of parents indicates that social support received predicts levels of stress significantly *p* < 0.001, meaning that social support received reduces parents’ stress levels (Table 7). Social support provided did not show significant results. When analysed according to sociodemographic and clinical variables as independent in the model, only the number of children was found to be significant *p* < 0.05. The size of the effect of social support received and the number of children over stress is low (*R*^2^ = 0.28), meaning that 28% of the variance is explained by the social support that parents receive and the number of children they have.

**Table 7:** Multiple linear regression analysis for stress, life and family satisfaction (*n* = 112).

Model	Non-standardised coefficients		Standardised coefficients	*t*	*p*
	*B*	*Standard error*	*Beta*		
**Stress**					
(constant)	4.854	0.642		7.563	0.000^**^
Gender of the parent/guardian	0.113	0.128	0.080	0.886	0.378
Age of parent/guardian	−0.004	0.011	−0.043	−0.375	0.709
Number of children	−0.200	0.096	−0.189	−2.089	0.039^*^
Gender of the child with cancer	0.216	0.123	0.160	1.754	0.083
Age of child with cancer	0.002	0.019	0.015	0.110	0.913
Children or AYAs	−0.433	0.244	−0.211	−1.775	0.079
Diagnosis time	−0.014	0.127	−0.010	−0.107	0.915
Social support received	−0.372	0.116	−0.375	−3.198	0.002^**^
Social support provided	−0.028	0.109	−0.029	−0.254	0.800
*R* = 0.528, *R*^2^ = 0.279, *R*^2^ adjusted = 0.212, *F* = 4.176, Sig = 0^**^					
**Life satisfaction**					
(constant)	2.244	1.169		1.920	0.058
Gender of the parent/guardian	−0.202	0.231	−0.081	−0.871	0.386
Age of parent/guardian	0.000	0.020	0.001	0.010	0.992
Number of children	0.086	0.162	0.049	0.534	0.595
Gender of the child with cancer	−0.271	0.221	−0.115	−1.228	0.223
Age of child with cancer	−0.006	0.035	−0.026	−0.177	0.860
Children or AYAs	0.159	0.447	0.044	0.357	0.722
Diagnosis time	−0.404	0.230	−0.166	−1.759	0.082
Social support received	−0.017	0.212	−0.009	−0.078	0.938
Social support provided	0.693	0.197	0.409	3.508	0.001^**^
*R* = 0.467, *R*^2^ = 0.218, *R*^2^ adjusted = 0.147, *F* = 3.060, Sig = 0.003^**^					
**Family satisfaction**					
(constant)	1.583	1.341		1.180	0.243
Gender of the parent/guardian	0.583	0.274	0.287	2.127	0.038
Age of parent/guardian	−0.020	0.022	−0.139	−0.902	0.371
Number of children	0.040	0.171	0.029	0.236	0.815
Gender of the child with cancer	0.330	0.226	0.179	1.459	0.151
Age of child with cancer	−0.006	0.034	−0.032	−0.182	0.856
Children or AYAs	−0.186	0.508	−0.062	−0.365	0.716
Diagnosis time	0.002	0.237	0.001	0.007	0.994
Social support received	0.403	0.204	0.328	10.977	0.050^*^
Social support provided	0.059	0.185	0.049	0.316	0.753
*R* = 0.546, *R*^2^ = 0.299, *R*^2^ adjusted = 0.175, *F* = 2.411, Sig = 0.023^*^					

* *p* ≤ 0.05;

** *p* ≤ 0.01.

*95% CI for the B. Gender: 1 = male and 2 = female; Children or AYA: 1 = 0–14 age and 2 = 15 age and over; Diagnosis time: 1 = 0–2 years and 2 = 3 years and over*.

**Figure 2:**
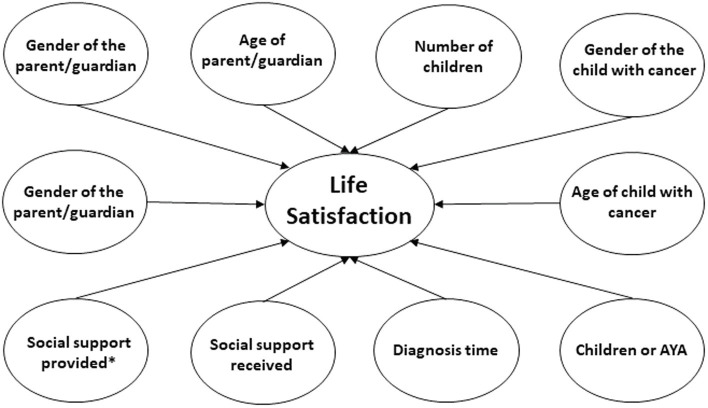
Life Satisfaction model.

In the second model analysed, life satisfaction was considered dependent variable and social support received and provided and sociodemographic and clinical variables were considered independent (Figure 2).

In this case, social support provided showed the most significant results *p* = 0.001, meaning that social support provided increased life satisfaction. The size of the effect of the model is *R*^2^ = 0.22, meaning that 22% of the variance is explained by social support provided. Social support received and the remaining sociodemographic and clinical variables did not predict life satisfaction. These results can be seen in Table 7.

**Figure 3:**
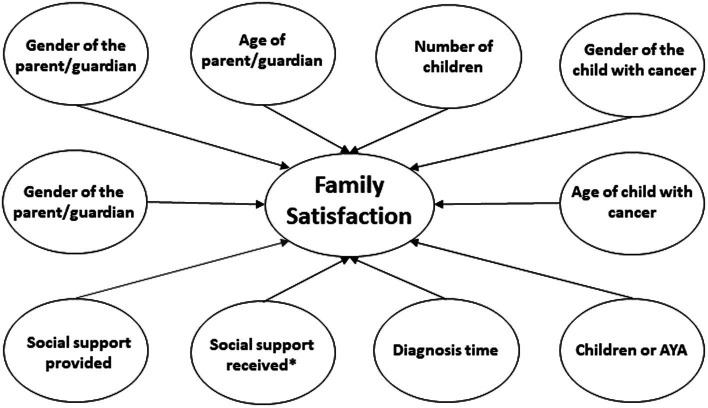
Family Satisfaction model.

In the third model, family satisfaction was considered dependent variable and social support received and provided and sociodemographic and clinical variables were considered independent (Figure 3). Social support received explains family satisfaction (*p* = 0.50), as it increases the family satisfaction of parents of children with cancer. The size of the effect of social support received on family satisfaction is *R*^2^ = 0.30, el 30% de la varianza es explicada por el apoyo social recibido. Social support provided and the remaining sociodemographic and clinical variables were not statistically significant (Table 7).

## Discussion

Most relevant results from this research show that male parents feel more satisfied with social support received and provided; however, it is female parents who feel higher family satisfaction. Parents of children aged 0–14 feel more stress than those parents of children aged 15–21. A positive and significant relation was found between satisfaction with social support received and provided and life and family satisfaction. However, a negative and significant relation between social support received and provided and stress was found. Additionally, social support received and parents’ number of children predict stress in parents, where higher satisfaction with social support received and higher number of children relate to lower levels of stress. On the other hand, social support provided by parents is the one that relates to life satisfaction, where higher satisfaction with social support provided shows higher life satisfaction. Finally, satisfaction with social support received relates to higher levels of family satisfaction.

Analyses conveyed emphasise the importance of knowing how the two dimensions of social support and sociodemographic and clinical variables affect stress levels as well as life and family satisfaction in parents of children with cancer. Most research on social support mainly focuses on analysing social support received. More specifically, studies have shown that support is a source that helps parents cope with the difficult situations caused by childhood cancer (Haunberger et al., 2019). However, social support provided by parents to other people facing the same situation is also a key variable to cope with such a difficult situation, as confirmed by the results from the present study.

In general, the hypotheses suggested were confirmed. The significant and negative relation between the two dimensions of social support and stress seems rather clear, as well as the significant and positive relation between social support received and provided and life and family satisfaction. When the effect of social support received and provided on the studied variables is analysed more in-depth results obtained are worth noting. Social support received is the variable that better predicts a decrease in stress levels, as well as higher family satisfaction. These results confirm the importance of receiving support to cope with daily family tasks as well as facing the different situations derived from the child’s disease in a more efficient manner (Choi et al., 2016). However, the increase of life satisfaction is generated by the social support provided by parents to other people, not by the social support received. This might be explained by the fact that on many occasions, those parents who provide support become examples for others (Hombrados-Mendieta et al., 2004). This fact may give parents back some control over their own lives, thus becoming active subjects in the development of resources, facing problematic situations and providing support (Hombrados-Mendieta and Martimportugués, 2006). This phenomenon may directly affect parents’ life satisfaction as they may feel they are able to help others thus feeling useful and turning their personal experiences into a potential way of helping themselves and others.

It is important to note that some sociodemographic and clinical variables are key to understand the object of study. There are gender differences in satisfaction with social support received and provided, where male parents feel more satisfied than female parents. This fact is relevant since it could be related to the roles in providing care, that tend to be done by women, who usually play the role of main carers (Cueto et al., 2013). Meaning that they might require higher support, therefore reducing their satisfaction. In this sense, it is also interesting to note that female parents feel higher family satisfaction. Family satisfaction relates to the system, connections and communication that take place between the family members (Sobrino, 2008); female parents might value more family union and communication dynamics that happen within the family. Parents of adolescents and young adults aged 15–21 (AYA) experience less stress than those parents of children aged 0–14. This result might be caused by the load of care that an ill child requires, since younger children are more dependent on their parents’ care, compared to AYAs, who also require care but are more autonomous in many daily chores. This fact might have relevant implications in parents’ perception of stress. It is also of interest to mention that the number of children is a variable that relates with stress perception. This might be due to the fact that having other children in the family means additional sources of support for parents, which might have a positive effect on parents’ perception of stress.

We would also like to highlight the fact the results obtained could be of relevant guidance towards achieving a better understanding on the relations between the study’s variables, despite the fact that the size of the effect is not very high that the sample studied is very specific, the low incidence in the general population and the fact that studying variables, such as social support provided and life and family satisfaction in parents of children and AYAs with cancer, is considerable new.

### Practical Implications

Some guidelines for intervention can be obtained from these findings. Given that support received related to lower levels of stress and higher family satisfaction, intervention guidelines for the family environment could be designed in order to provide parents with the support they need. Individuals usually receive support from their natural networks of support, which are mainly formed by relatives and friends. These natural networks provide a wide range of types of support. However, as noted by Hombrados (2013), sometimes these networks are willing to provide help, but they do not know how. Very often, it is necessary to work with relatives or the closest support network of those who are facing the problematic situation. This is often the case in individuals who suffer from cancer and other severe diseases, as such diseases can alter the support network and feelings of fear or avoidance might appear in the members of support networks. Not knowing which is the appropriate way of taking care of those parents of children with cancer can lead to anxiety. Undesired and opposed effects to the reception of support may give rise to physically avoiding the ill person or avoiding communication about the disease. It is necessary, therefore, to intervene and assess the needs of parents and develop competencies in the provision of help by their closest support network.

Regarding support provided, it has been confirmed that parents feel higher life satisfaction when they provide support to others. Keeping themselves active, helping other parents or participating in associations is likely to make them have active coping mechanisms to face their child’s disease. In this sense, intervention guidelines could be designed in order to reduce the helplessness feelings caused by their child’s situation through participating and promoting the empowerment of parents.

Sociodemographic and clinical variables of families, the role of other children in the family, the ages of children with cancer (children and AYAs) and the gender of parents should be further explored and studied, since they are tightly linked to the role undertaken by parents as main carers.

Having a broader understanding of the effect of social support received and provided and sociodemographic and clinical variables on stress levels and life and family satisfaction can have highly relevant implications in the design of intervention guidelines that improve parents’ life situations. It seems clear that improving parents’ satisfaction requires addressing psychosocial aspects (Sánchez-Egea et al., 2019), for which it is necessary to know how these variables relate based on the families that face these situations (Kedia et al., 2020).

### Limitations

Among the limitations of the present study, its cross-sectional design is to be noted. For future research, it would be convenient to carry out a longitudinal design in order to better know the relations between variables and how they affect each other. Furthermore, all participants came from Malaga (Spain), which limits the extrapolation of findings to other contexts and cultures. It would be interesting to convey this study in other countries.

Regarding the features of the sample, there were more mothers than fathers, so it would be important to balance the number of mother and father participants to know their needs for support differently.

However, it is important to bear in mind that this fact reflects a social reality—the number of women who take care of their children is significantly higher than the number of men. Moreover, women tend to participate more in this kind of research (García-Calvente et al., 2004).

Other matters to be considered in future lines of research include considering other relevant sociodemographic variables, which might be related to stress management and life and family satisfaction of parents, such as the stage of cancer where children are and the different types of cancer. On the other hand, studying the reciprocity and differences between social support received and provided in future lines of research could also be of interest to find out if such differences are also relevant in coping with cancer in children. Other limitation to consider is the common source bias, which means that the fact that parents could belong to the same family unit in some cases was not controlled. Information on those parents who did not wish to participate was not gathered either, which can also imply a limitation in the generalisation of results.

## Conclusion

One of the strengths of this study is that novel contributions have been made on the relations between variables related to the process of facing childhood and young adulthood cancer by parents. To broaden the knowledge on these relations, it is key for parents to adapt to the situation caused by the disease, clinical practices and action plans. There are very few quantitative studies that focus on studying the psychosocial variables of parents in the context of childhood and young adulthood psycho-oncology.

It is necessary to consider the practical relevance of the findings from the present study and apply them to the daily tasks of those professionals who provide psychosocial support in these situations. Some of the therapeutic strategies that could be applied by professionals is to know and promote parents’ social support networks and know the level of social support they receive and provide. This can help reduce the negative effects of stress and increase levels of life and family satisfaction.

## Data Availability Statement

The raw data supporting the conclusions of this article will be made available by the authors, without undue reservation.

## Ethics Statement

The studies involving human participants were reviewed and approved by the Comité Ético de la Junta de Andalucía. The patients/participants provided their written informed consent to participate in this study.

## Author Contributions

IH-M and MM-M: conceptualization and methodology. AM-G and MM-M: formal analysis and writing—original draft preparation. AM-G, IH-M, MM-M, and IR-R: investigation, writing—review and editing, and supervision. IH-M: funding acquisition. All authors have read and agreed to the published version of the manuscript.

## Funding

This work was supported by the Groups PAIDI, Junta de Andalucía, Spain [grant no. HUM-590].

## Conflict of Interest

The authors declare that the research was conducted in the absence of any commercial or financial relationships that could be construed as a potential conflict of interest

## Publisher’s Note

All claims expressed in this article are solely those of the authors and do not necessarily represent those of their affiliated organizations, or those of the publisher, the editors and the reviewers. Any product that may be evaluated in this article, or claim that may be made by its manufacturer, is not guaranteed or endorsed by the publisher.
